# Monte Carlo-based QSPR modeling of drop-weight impact sensitivity of energetic materials using hybrid SMILES–graph descriptors

**DOI:** 10.1039/d6ra07617c

**Published:** 2026-10-14

**Authors:** Shahram Lotfi, Shahin Ahmadi, Parvin Kumar

**Affiliations:** a Department of Chemistry, Payame Noor University (PNU) 19395-4697 Tehran Iran Sh.lotfi@pnu.ac.ir Sh.Lotfei@gmail.com; b Department of Chemistry, TeMS.C., Islamic Azad University Tehran Iran ahmadishahin@iau.ac.ir ahmadi.chemometrics@gmail.com; c Department of Chemistry, Kurukshetra University Kurukshetra Haryana 136119 India

## Abstract

Impact sensitivity under mechanical stimuli is one of the most crucial safety properties of energetic materials. The drop-weight test is a common method for evaluating the impact sensitivity of explosives, with results typically reported as the 50% initiation height (*H*_50_) or drop energy (*E*_50_ (cm)). In this study, three quantitative structure–property relationship (QSPR) models were developed and assessed to predict the logarithm drop-weight impact sensitivity (log *E*_50_) of various types of explosive materials using a Monte Carlo algorithm implemented *via* CORAL software. The descriptor of correlation weight (DCW) was computed from the SMILES notations of the compounds. The correlation intensity index (CII) and the index of ideality of correlation (IIC) were employed as supplementary parameters for optimizing the correlation weights (CW) utilized in the computation of optimal descriptors. A dataset of 515 explosives or energetic compounds was employed to make QSPR models utilizing hybrid optimal descriptors derived from SMILES and hydrogen filled graph (HFG). The dataset was partitioned into four subsets: active training, passive training, calibration, and validation. The prediction ability of generated models was evaluated by various validation criteria. For the log *E*_50_ endpoint, the models obtained across three independent random data splits achieved an average validation-set *R*^2^ of 0.7005 ± 0.0094. An average determination coefficient of 0.7162 ± 0.0129 was obtained for the active training sets. The structural attributes that promote an increase/decrease in log *E*_50_ were extracted and discussed, providing a mechanistic interpretation of the model.

## Abstract 2

A hybrid SMILES–graph descriptor enables prediction of drop-weight impact sensitivity for 515 energetic compounds using a compact CORAL-based QSPR approach.

## Graphical abstract

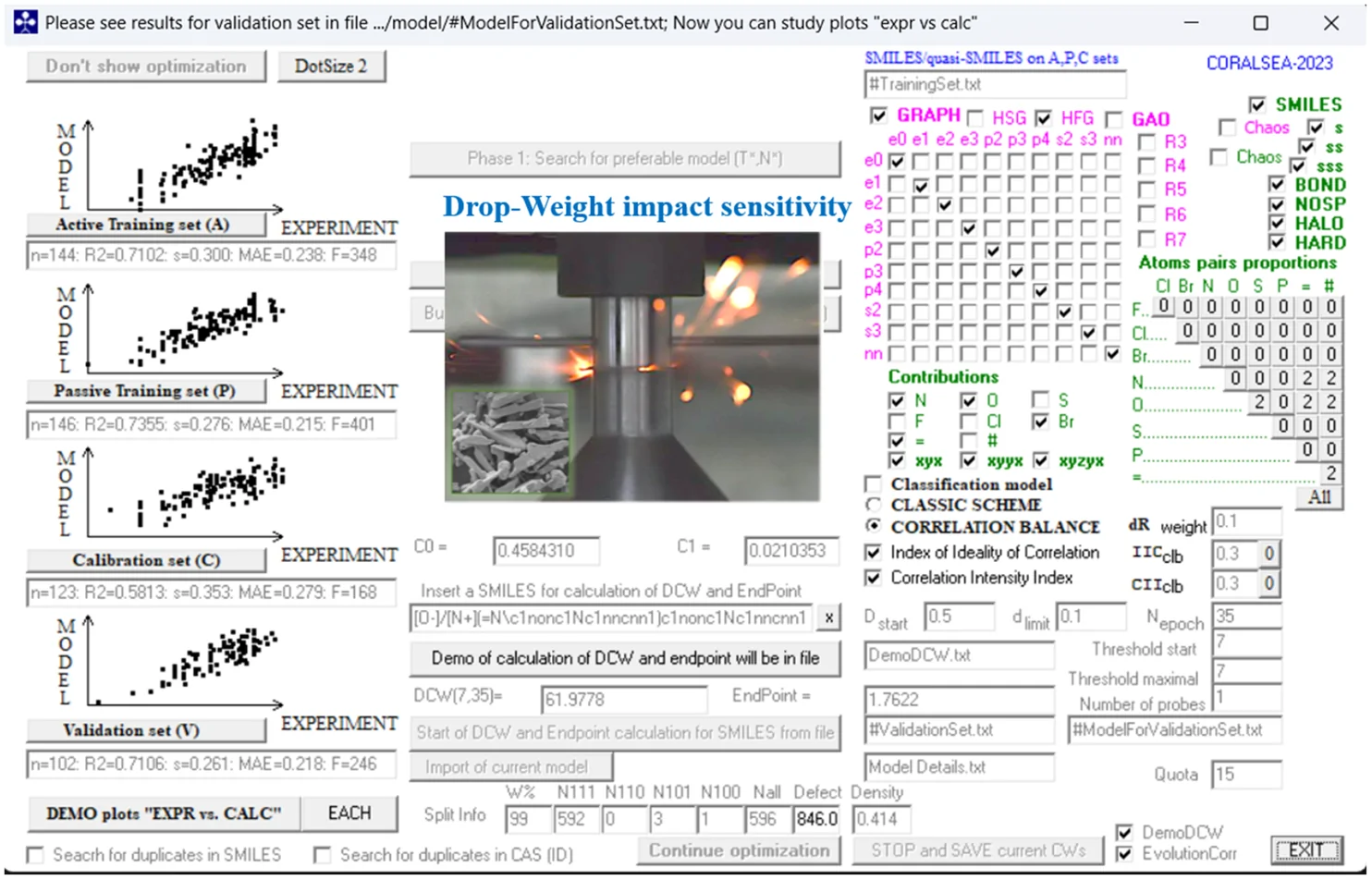

## 1. Introduction

Energetic materials, including explosives, are widely used in various fields of industry and defense. During production, storage, and handling, these materials are exposed to various stimuli such as impact, heat, spark, shock and friction, any of which may initiate detonation. Among these, impact sensitivity is particularly significant because it directly relates to the safety and handling of energetic compounds and is commonly expressed as the 50% initiation height (*H*_50_) or drop energy (*E*_50_). This property is often employed to compare the safety of explosives and is always a meaningful research topic. Normally, to determine impact sensitivity, the drop-weight impact test is applied. Experimentally, a milligram scale (typically ≈ 40 mg) of an energetic material is placed between a flat steel anvil and a striker. A weight, typically 2.5 kg, is then dropped from a specified height onto the striker plate, and the occurrence or absence of a reaction is documented. Outcomes are published as the height (or energy) at which 50% of the trials result in an explosion, *i.e.*, the *H*_50_ (or *E*_50_) value. So, explosives with higher *H*_50_ values have lower impact sensitivity and thus more safety.^1–5^ However, experimental drop-weight tests are often time-consuming, expensive, and hazardous, and the results can be irreproducible, with widely varying *H*_50_ values reported for the same compound.^6,7^

Computational approaches, such as quantitative structure–property relationships (QSPR) modelling, offer a viable alternative technique for predicting various physicochemical properties utilizing descriptors derived only from the molecular structure.^8,9^ QSAR modelling is a mathematical approach that establishes correlations between endpoints (activity/property/toxicity) and structural information of chemicals (2D and 3D).^10,11^ In the case of energetic materials, some QSPR approaches have been already employed to assess the impact sensitivity, most of them are for nitro compounds.^9,12–15^

Early work by Kamlet in 1976 (ref. 16) reported linear models relating log *H*_50_ to the oxygen balance with high correlations. Nefati *et al.*^17^ in 1996 established a QSPR model to predict the impact sensitivity of 204 molecules using thirteen molecular descriptors and artificial neural network (ANN). Later, in 2005, Cho *et al.*^18^ optimized the NN architecture based on Nefati's work, using 263 energetic molecules and 10–20 molecular descriptors for different QSPR model.

In 2009, Wang *et al.*^2,13^ developed QSPR models for 156 and 186 nitro compounds using electrotopological-state indices (ETSI) combined with back propagation neural network (BPNN), multiple linear regression (MLR), and partial least squares (PLS). Morrill and Byrd^12^ in 2008 built QSPR based for 227 nitro compounds using AM1 semi-empirical quantum mechanical descriptors and robust least median squares regression.

Xu *et al.*^9^ in 2012 proposed QSPR models for 156 energetic compounds based on 10 3D descriptors, using MLR for linear models and ANN for nonlinear models. In 2016, Mathieu^19^ developed QSPR based on physical assumptions, combining data from Fayet *et al.*^14^ and Prana *et al.*^8^ for 60 N-NO_2_ and 50 C-NO_2_ compounds, respectively.

Previous studies have applied both conventional QSPR and machine-learning-based methods to the prediction of impact sensitivity. These approaches have included multiple linear regression, partial least-squares regression, artificial neural networks, and models based on three-dimensional, physicochemical, or quantum-chemical descriptors.^2–4,7–9,12–15^ Machine-learning methods can represent complex and nonlinear relationships between molecular characteristics and impact sensitivity, particularly when sufficiently informative descriptors and representative datasets are available. However, their performance may depend strongly on the size and chemical diversity of the dataset, descriptor selection, model architecture, hyperparameter optimization, and validation strategy. In addition, highly flexible models may be more difficult to interpret mechanistically and may be susceptible to overfitting when the number of structural variables is large relative to the number of available compounds.

In contrast, structure-based QSPR approaches using compact descriptors can provide a more computationally accessible representation of molecular information and may facilitate the interpretation of structural contributions. However, simpler QSPR models may not capture all nonlinear or complex relationships that can be represented by advanced machine-learning algorithms. Consequently, the selection between QSPR and machine-learning approaches should depend on the purpose of the study, the available structural information, dataset characteristics, validation requirements, and the desired balance between predictive performance, computational complexity, and interpretability.

The CORAL (CORrelation And Logics) program has been recommended as a useful tool for constructing QSPR/QSAR models using input data lists of SMILES (Simplified Molecular Input Line Entry System) strings of the molecules, together with corresponding experimental data on endpoints. The Monte Carlo method is employed to build models using CORAL software.^20–22^ This approach has been successfully applied to predict a wide range of properties, including physicochemical, pharmacological, toxicological, and ecological.^23–27^

From a methodological perspective, CORAL provides a structure-based QSPR framework in which molecular information extracted from SMILES notation and molecular graphs is converted into correlation weights and combined to construct an optimal descriptor. Unlike descriptor-intensive approaches that require a large predefined set of physicochemical, quantum-chemical, or three-dimensional descriptors, CORAL can use two-dimensional structural representations as the basis for descriptor generation, which is advantageous for structurally diverse datasets where consistent three-dimensional or quantum-chemical descriptors are computationally demanding. The correlation weights are optimized using a Monte Carlo procedure, directly connecting descriptor construction to a selected statistical target function and permitting the incorporation of additional criteria such as the index of ideality of correlation (IIC) and the correlation intensity index (CII). Nevertheless, CORAL should be regarded as a complementary QSPR methodology rather than a universally superior alternative to other statistical or machine-learning approaches; its suitability here was therefore examined using multiple data splits, calibration-based optimization, external validation, and model-randomization analysis.

The objective of the present research is to evaluate the CORAL software as a tool for building QSPR models to estimate the log *E*_50_ (“drop energy”) of 515 explosives or energetic compounds using the built-in Monte Carlo algorithm. Here, two-dimensional hybrid descriptors are used to reduce computational complexity. The predictive ability of the generated models is evaluated using internationally accepted parameters (internal and external validations, model randomization), and a mechanistic interpretation of the structural features influencing is provided. The index of ideality of correlation (IIC) and the correlation intensity index (CII) criteria are used to assess the predictive potential of the QSPR models.

## 2. Method

### 2.1. Data

In the present QSPR model development study, the prediction of drop-weight impact sensitivity was described for 515 diverse energetic compounds. The log *E*_50_, along with SMILES notations of energetic compounds was taken from the article published by Marrs *et al.*^28^

The original dataset reported by Marrs *et al.* comprised 1533 experimental observations corresponding to 515 unique molecules. Prior to model development, the SMILES representations were standardized and canonicalized using RDKit (version 2026.03.6), including molecular normalization, uncharging, selection of the largest molecular fragment, and generation of canonical SMILES. This procedure provided a consistent representation of the molecular structures and functional groups, including nitro groups, and minimized variations arising from alternative SMILES encodings. For molecules with repeated measurements, the arithmetic mean of the reported experimental values was calculated, and the resulting average was used as the representative log *E*_50_ value. Consequently, the final dataset comprised 515 unique molecules, each represented by a single averaged experimental log *E*_50_ value. The number of replicate measurements, averaged log *E*_50_ values, standard deviations, and ranges for the molecules with repeated measurements are provided in the (Table S1). The endpoint log *E*_50_ was taken as the dependent parameter for constructing the QSPR models, and all subsequent data splitting and model development were performed at the molecular level. To assess the robustness of the QSPR approach, three random splits were generated. Each split was randomly divided into four subsets: active training (≈29%), passive training (≈26%), calibration (≈25%) and validation (≈20%) set. The purpose of each set in generating the QSPR models has been reported in the literature.^29,30^

The percentage identity of splits was also computed and is demonstrated in Table 1, confirming that the three splits are distinct, with no pair of splits sharing more than 29.0% identical distributions. The detailed dataset, including chemical IDs, SMILES notation, subset assignments, experimental and predicted log *E*_50_ values, and applicability domain information for all splits, is provided in the (Table S2).

**Table 1:** The percentage of identical distributions on three considered splits

Split	Set	Split 1	Split 2	Split 3
1	Active training	100	26.5	21.8
	Passive training	100	28.3	23.2
	Calibration	100	22.7	24.3
	Validation	100	29.0	20.6
2	Active training		100	22.5
	Passive training		100	24.6
	Calibration		100	25.8
	Validation		100	25.3
3	Active training			100
	Passive training			100
	Calibration			100
	Validation			100

### 2.2. CORAL model

The QSPR model for log *E*_50_ created by CORAL has the following general form:

1 log *E*_50_ = *C*_0_ + *C*_1_ × DCW(T*, N*)

Here, *C*_0_ and *C*_1_ are regression coefficients calculated using the least squares method, and DCW(T*, N*) is the hybrid optimal descriptor computed through the correlation weights of molecular features extracted from SMILES and molecular graphs. In the CORAL program, the Monte Carlo optimization method was used to compute the correlation weights that maximize a chosen target function.

The theoretical basis of the CORAL approach is that molecular structure can be represented by a collection of structural attributes derived from SMILES notation and molecular graphs. Each structural attribute is assigned a correlation weight (CW), and the weighted contributions of the structural attributes are combined to calculate a hybrid optimal descriptor. In the present study, the descriptor includes information obtained from both SMILES and Hydrogen filled graph (HFG) representations. The use of these two representations was intended to incorporate complementary structural information, including local molecular patterns and graph-based connectivity features.

The Monte Carlo algorithm in CORAL is used as an optimization procedure for determining the correlation weights associated with the structural attributes. The optimization begins with an initial set of correlation weights. During each Monte Carlo step, selected weights are modified, and the resulting descriptor is recalculated for the compounds in the relevant data subsets. The descriptor is then used in a regression equation relating the calculated descriptor values to the experimental log *E*_50_ values. The quality of the resulting model is evaluated using the selected target function. Changes in the correlation weights are retained when they improve the target function according to the optimization criterion, whereas changes that do not improve the objective are not accepted or are treated according to the optimization rules implemented in CORAL.

The main role of the Monte Carlo algorithm in the present study was therefore not simply to fit the regression coefficients, but to optimize the structural correlation weights used to generate the hybrid descriptor. The regression coefficients *C*_0_ and *C*_1_ were subsequently calculated using the least-squares method. This distinction is important because the descriptor-generation process and the regression step represent two related but separate stages of the QSPR modeling procedure.

In this procedure, the threshold parameter *T* controls the range or conditions associated with the optimization of the correlation weights, whereas the number of epochs *N* defines the number of repeated optimization cycles. Different combinations of *T* and *N* are examined, and the corresponding models are evaluated using the calibration subset. The combination denoted as T* and N* is selected according to the best calibration performance for the specified target function. The final descriptor, DCW(T*, N*), is then used to construct the QSPR model.^25,31,32^ In this work, a range of 1–15 for *T* and 1 to 40 for *N* was explored to identify the best T* and N*.

### 2.3. Hybrid optimal descriptor

A hybrid optimal descriptor was used to generate the QSPR model of log *E*_50_. This descriptor was obtained by the amalgamation of molecular attributes extracted from SMILES and hydrogen filled graph (HFG).

To calculate a hybrid optimal descriptor applying the CORAL program, following mathematical equation is used:

2 ^Hybrid^DCW(T*, N*) = DCW_SMILES_(T*, N*) + DCW_HFG_(T*, N*)

3 ^SMILES^DCW(T*, N*) = ∑CW(S_K_) + ∑CW(SS_K_) + ∑CW(SSS_K_) + CW(NOSP) + CW(HALO) + CW(HARD) + CW(BOND) + CW(*xyx*) + CW(*xyyx*) + CW(*xyzyx*)

4 ^HSG^DCW(T*, N*) = ∑CW(EC_0k_) + ∑CW(EC_1k_) + ∑CW(EC_2k_) + ∑CW(EC_3k_) + ∑CW(Ep2_k_) + ∑CW(Ep3_k_) + ∑CW(Ep4_k_) + ∑CW(VS2_k_) + ∑CW(VS3_k_) + ∑CW(nn_k_)

The detailed description of SMILES and graph attributes invariants used in eqn (3) and (4) is provided in Table 2. The term CW(*X*) indicates the correlation weight for the SMILES and HFG attributes, as defined in Table 2.

**Table 2:** Detailed description of the SMILES and graph attributes used in eqn (3) and (4)

Attribute	ID	Definition
SMILES attribute	Sk	SMILES atom, *i.e.*, one symbol (*e.g.* ‘C’, ‘N’, ‘=’, *etc.*) or a group of symbols that cannot be examined separately (*e.g.*, ‘Cl’, ‘Br’, Si’, *etc*)
	SSk and SSSk	Local SMILES attributes described as a combination of two and three SMILES-atoms
	BOND	Presence or absence of chemical bonds: double (=), triple (#), and stereochemical (@) or @@)
	HALO	Existence or absence of fluorine (F), chlorine (Cl), and bromine (Br)
	HARD	Association of BOND, NOSP, and HALO in the united structural code
	NOSP	Global SMILES features and it denotes the presence or absence of different chemical elements: nitrogen (N), oxygen (O), sulfur (S), and phosphorus (P)
	*xyx*, *xyyx*, and *xyzyx*	Fragments of local symmetry (FLS), the *x* and *y* are one-symbol SMILES atoms, and these obey the following rules: *x* ≠ *y* and *y* ≠ *z*. They take into consideration a local, focused moiety of the molecule and not the global symmetry.-
HFG features	E0k	Vertex degree
	E1k, E2k and e3k	Morgan extended connectivity of first, second and third orders
	pt2k, pt3k and pt4k	Number of paths of lengths 2, 3 and 4 beginning from a given vertex in the HFG
	S2_k_ and S3_k_	Valence shells of the second and third orders
	nnk	nearest neighbours code

### 2.4. Optimization of correlation weights

Correlation weights (CW) are optimized using the Monte Carlo algorithm with active training, passive training, and calibration sets. Initial CW values for molecular attributes were randomly selected and iteratively replaced; changes were accepted only when the target function improved.

The target function defined the statistical objective for CW optimization.

The optimization strategy adopted in this study incorporates two additional criteria, namely the index of ideality of correlation (IIC) and the correlation intensity index (CII).^33^ These criteria are introduced to direct the stochastic optimization toward those regions of the training dataset that provide more transferable information and are therefore more relevant to the calibration subset. The contribution of IIC and CII to the optimization process is controlled through their corresponding weighting coefficients. The weights are selected empirically by examining the behavior of the stochastic optimization procedure under different combinations of IIC and CII contributions.

Accordingly, the target function used in the present study is defined as follows:

5 TF_3_ = *R*_ATRN_ + *R*_PTRN_ − |*R*_ATRN_ − *R*_PTRN_| × 0.1 + (IIC_CAL_ + CII_CAL_) × 0.3

Here, *R*_ATRN_ and *R*_PTRN_ are the determination coefficients between the predicted and observed log *E*_50_ values for the active and passive training sets, respectively.

The Index of Ideality Correlation (IIC) for the calibration set is calculated as:6
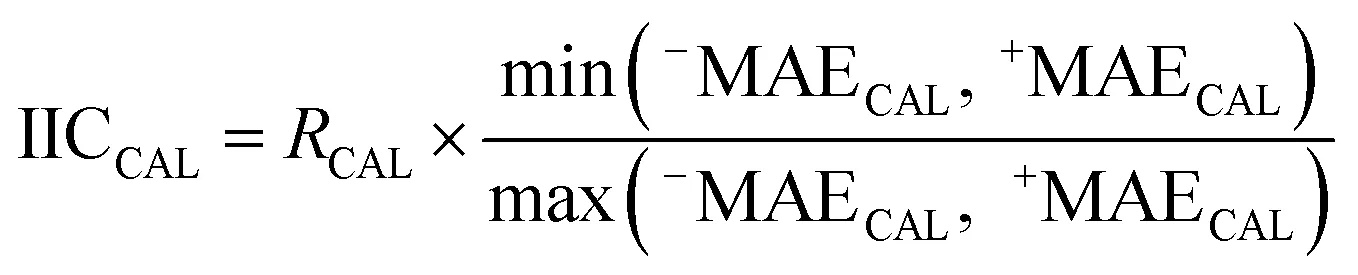
Here, *R*_CAL_ is the determination coefficient between observed and calculated values of log *E*_50_ for the calibration set. The ^−^MAE and ^+^MAE are indicate negative and positive mean absolute errors, defined as:7
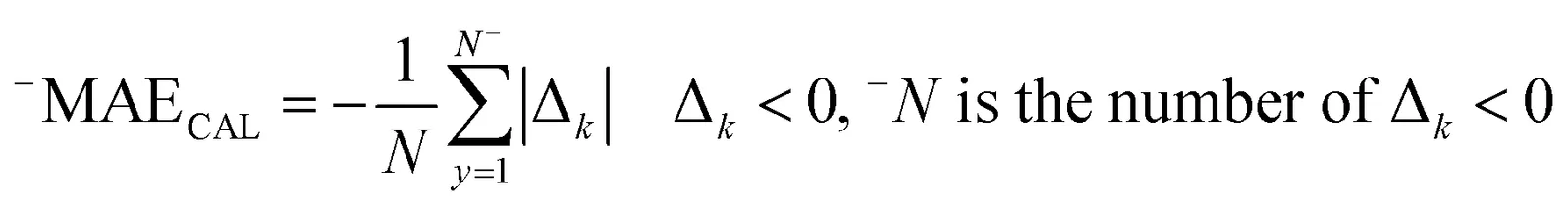
8
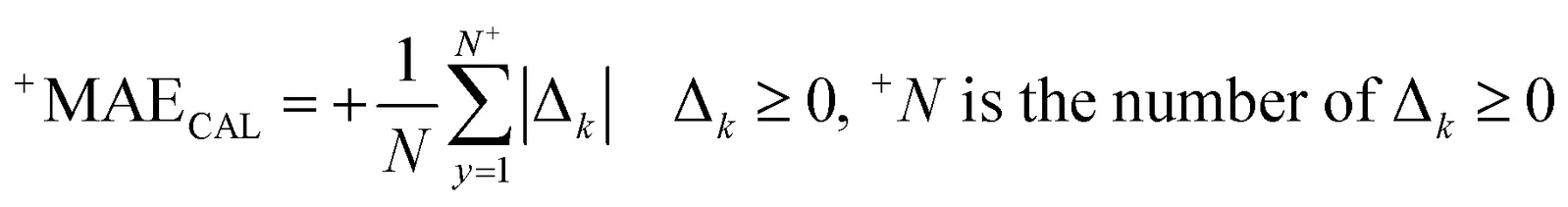
Here, the ‘*k*’ is the index (1, 2, … *N*) ,Δ_*k*_ explains the predictive ability for a substance in any set and is defined as follows:

9 Δ_*k*_ = observed_*k*_ − calculated_*k*_

The observed_*k*_ and calculated_*k*_ are related to the endpoint.

The CII same as IIC, has been employed to improve the quality of the Monte Carlo optimization to develop QSPR/QSAR models.^34,35^ The CII was calculated using data on the calibration set as follows:10
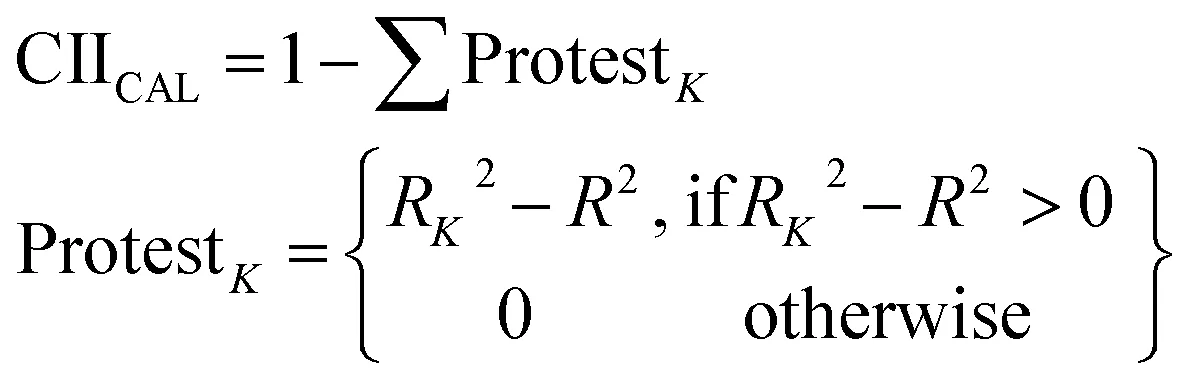


The *R*^2^ is the correlation coefficient for a set that contains *n* substances. The *R*_*K*_^2^ is the correlation coefficient for *n*−1 substances of a set after removing of *K*th substance. Hence, if the (*R*_*K*_^2^ − *R*^2^) is more than zero, the *K*th substance is an “oppositionist” for the correlation between experimental and predicted values of the set. A small sum of “protests” means a more “intensive” correlation.

## 3. Results and discussion

### 3.1. QSPR modelling and validation

To ensure the statistical robustness and reliability of the resulting models, three QSPR models was established using the Monte Carlo optimization procedure. Model development was based on a hybrid optimal descriptor that integrates information encoded in SMILES notation with structural information derived from the HFG representation. To achieve maximum prediction accuracy for each split, the threshold (*T*) and number of epochs (*N*_epoch_) were varied in the ranges 1–15 and 1–40, respectively.

For each of the three random partitions of the dataset, the QSPR models were developed using four subsets, namely the active training, passive training, calibration, and validation sets, as presented below:

11 split-1: log *E*_50_ = 0.4584 (0.0034) + 0.0210 (0.0001) × DCW(7,35)

12 split-2: log *E*_50_ = 0.6000 (0.0026) + 0.0208 (0.0001) × DCW(7,35)

13 split-3: log *E*_50_ = 0.6187 (0.0025) + 0.0206 (0.0001) × DCW(7,35)

Table 3 summarizes the statistical characteristics of the log *E*_50_ models developed for the investigated explosives using eqn (10)–(13).

**Table 3:** Statistical performance of the models obtained from three random dataset partitions into active training (A), passive training (P), calibration (C), and validation (V) subsets

Split	Set	*n*	*R* ^2^	CCC	IIC	CII	*Q* ^2^	*Q*_F1_^2^	*Q*_F2_^2^	*Q*_F3_^2^	RMSE	MAE	*F*	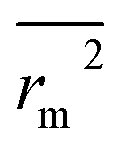	Δ*r*_m_^2^	Y-test	5-Fold	R*m^2^ (test)	*r*_m_^2^
1	ActivTRN	151	0.7102	0.8306	0.8197	0.8549	0.7028				0.300	0.238	348				0.7025		
	Pass TRN	134	0.7355	0.8329	0.7224	0.8663	0.7281				0.276	0.215	401				0.7295		
	Calib	128	0.5813	0.709	0.7624	0.7962	0.5685	0.5716	0.5693	0.5802	0.353	0.279	168	0.4036	0.305		0.5648	0.4036	0.5512
	Valid	102	0.7106	0.8252	0.6803	0.8402	0.6984				0.2613	0.2184	246	0.6021	0.1714	0.0099	0.6954	0.6878	0.5164
2	ActivTRN	153	0.7073	0.8286	0.8308	0.8523	0.7011				0.304	0.239	389				0.7008		
	Pass TRN	147	0.7585	0.8368	0.7118	0.8723	0.7524				0.284	0.223	471				0.7527		
	Calib	112	0.6299	0.7916	0.7936	0.8165	0.6172	0.6099	0.6028	0.7054	0.303	0.241	170	0.5098	0.0339		0.6176	0.5098	0.5928
	Valid	103	0.6921	0.8311	0.7158	0.8367	0.6787				0.2787	0.2261	216	0.5857	0.059	0.0112	0.6796	0.6152	0.5562
3	ActivTRN	151	0.731	0.8446	0.7792	0.8488	0.7249				0.286	0.216	405				0.7251		
	Pass TRN	143	0.7636	0.8554	0.6748	0.8722	0.7565				0.259	0.202	455				0.7567		
	Calib	119	0.6373	0.7717	0.7983	0.8316	0.6256	0.6323	0.632	0.62	0.333	0.267	206	0.5096	0.244		0.6234	0.5096	0.5876
	Valid	102	0.6989	0.8044	0.7517	0.8113	0.6408				0.3013	0.2363	190	0.5405	0.0367	0.0085	0.6379	0.5221	0.5588

The simultaneous use of IIC and CII improved the predictive potential of models, especially the statistical quality of the validation set, at the expense of somewhat lower performance on the training set.^36,37^

The use of IIC and CII can be regarded as a form of “statistical forcing”: the correlation weights are optimized to be more favorable for the calibration set than for the training sets, with the expectation that this will also enhance external predictivity. This strategy encourages a more general model instead of one that fits the training data too closely, thereby reducing the risk of overfitting.

The results indicate that the models exhibit satisfactory predictive performance across all three random data splits, as reflected by the statistical parameters obtained for the validation subsets. In each split, the coefficient of determination for the validation set exceeds 0.60, with the highest value reaching 0.7106 for the first split. The average values of the coefficient of determination, together with their corresponding dispersions, were 0.7162 ± 0.0129, 0.7525 ± 0.0150, 0.61625 ± 0.0304, and 0.7005 ± 0.0094 for the active training, passive training, calibration, and validation sets, respectively. The mean absolute error (MAE), which provides an additional measure of predictive accuracy, varied between 0.202 and 0.279 across the developed models.

It has been shown that *r*_m_^2^(test) is a stricter measure of external predictivity than *R*_pred_^2^.^38^ An *r*_m_^2^ value greater than 0.5 is generally considered indicative of an acceptable level of model reliability. In this study, for all three data splits, the *r*_m_^2^ values obtained for the validation subsets exceeded the 0.5 threshold, indicating satisfactory predictive performance.

The scatter plot of experimental *versus* predicted log *E*_50_ values for the three models (Fig. 1A) shows good agreement between observed and calculated data. Residual plots of log *E*_50_*versus* experimental values for all subsets and all splits (Fig. 1B) reveal residuals scattered closely around the horizontal line at zero, indicating that the models are well fitted without obvious systematic bias.

**Fig. 1:**
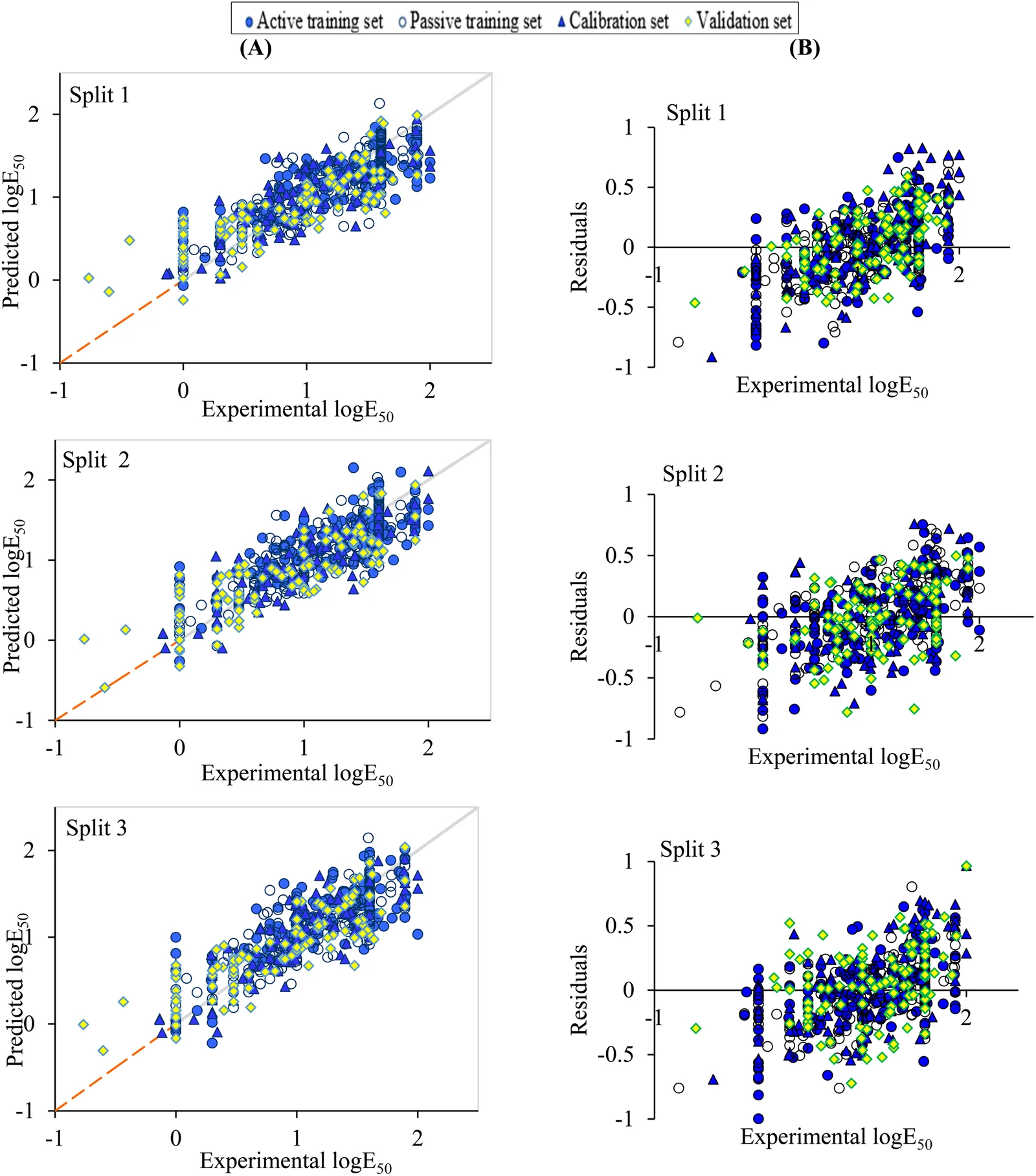
Graphical illustration of experimental *versus* calculated log *E*_50_ values (A) and residual of log *E*_50_*versus* experimental log *E*_50_ values (B) for three QSPR models.

### 3.2. Applicability domain of model

The applicability domain (AD) analysis allowed assessment of whether a given prediction was reliable based on the similarity of a compound to the training molecules. In this context, the AD was used to distinguish between predictions that fall within the reliable space of the model and those that are likely to be extrapolations.

For the CORAL models, the AD was defined using the statistical defects of SMILES attributes, calculated from their frequencies in the active training, passive training, and calibration sets (the validation set being external).^39–41^

The statistical defect for a given attribute *A*_K_computed as14
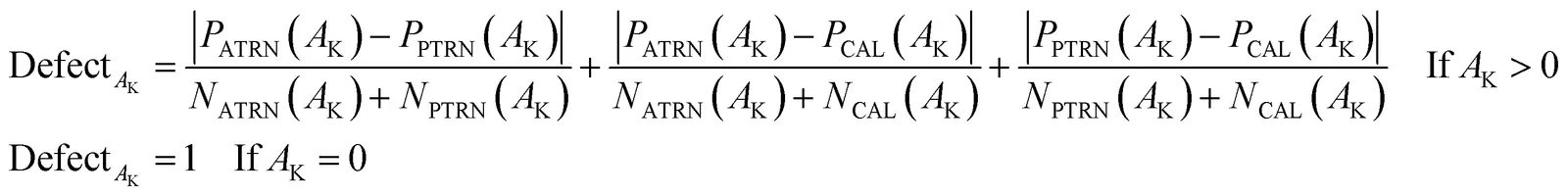


*P*_ATRN(*A*_K_)_, *P*_PTRN(*A*_K_)_ and *P*_CAL(*A*_K_)_ are the probability of attributes (*A*_K_) in the active, passive training sets, and calibration set, respectively; *N*_ATRN(*A*_K_)_, *N*_PTRN(*A*_K_)_, and *N*_CAL(*A*_K__, are frequencies of attributes (*A*_K_) in the active training, passive training and calibration sets, respectively.

The SMILES-statistical defect (*D*) can be calculated as the sum of statistical defects of all attributes.15
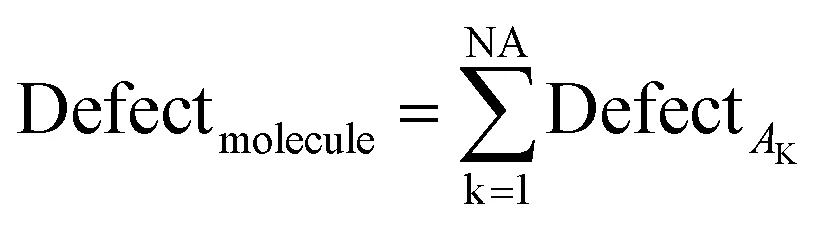
NA is the number of non-blocked SMILES attributes for the given compounds.

In CORAL, a SMILES is an outlier if:16
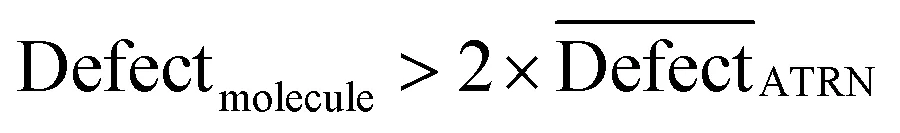

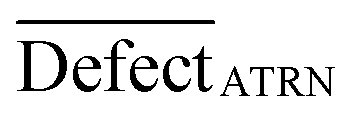
 is an average of statistical defects for the dataset of the active training set.

### 3.3. Model validation

Validation of the constructed QSPR models is essential for assessing their robustness and reliability. Several statistical criteria were used to evaluate the predictive potential of the models, following standard QSAR/QSPR practice. Internal validation utilizing the training datasets by measuring the determination coefficient (*R*^2^), root-mean-square error (RMSE), mean absolute error (MAE), cross-validated correlation coefficient (*Q*^2^), and matrices such as 
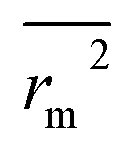
 and Δ*r*_m_^2^; 2). External validation utilizing the test datasets by calculation of statistical criteria such as concordance correlation coefficient (CCC), *Q*_F1_^2^, *Q*_F2_^2^, *Q*_F3_^2^, standard error of estimation (*s*), mean absolute error (MAE), Fischer ratio (*F*), root-mean-square error (RMSE) and), *r*_m_^2^(test) (*i.e.*
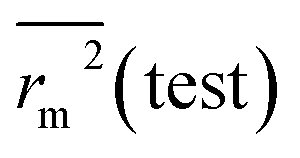
,Δ*r*_m_^2^(test));^38,42,43^ and 3) data randomization or Y-scrambling.^44^ In addition, Y-scrambling (data randomization) was performed to ensure that the models were not the result of chance correlations. IIC and CII were also used as final diagnostic parameters of predictive potential.^25,35,45^

The mathematical expressions for these validation metrics have been extensively reported in earlier studies^46–48^ and are not repeated here. Table 3 summarizes the statistical characteristics of the models for log *E*_50_ for all three splits. The results show good performance in both internal and external validation metrics. All generated QSPR models were statistically sound, and the values of the validation parameters fell within ranges considered acceptable in the QSAR/QSPR literature.^49,50^

### 3.4. Mechanistic interpretation

The mechanistic interpretation of QSPR models is a critical endeavor, and the CORAL software facilitates this process directly and efficiently. Monte Carlo optimization analyzes the calculated correlation weights (CW) for all structural attributes (SAk) across different data splits, enabling each attribute to be characterized individually. CWs(SAk) derived from both SMILES and graph-based descriptors categorize molecular features into three groups: (1) features that promote an increase in the endpoint (positive CW); (2) features that promote a decrease (negative CW); and (3) features with an undefined or inconsistent role (both positive and negative CW). These promoters provide valuable mechanistic insights into the QSPR models developed using CORAL's built-in Monte Carlo algorithm. Here, the most important structural features (SAk) that are recognized as promoters of increasing and decreasing the log *E*_50_ endpoint for the three models are introduced. Table 4 presents all structural attributes along with their correlation weights from three runs of the developed QSPR model for splits 1–3.

**Table 4:** List of promoters for the increase and decrease of log *E*_50_, along with their correlation weights in runs 1–3, the frequency of each attribute in each set, and examples of interpretative attributes

Split no.	SAk	CWs run 1	CWs run 2	CWs run 3	*N*_AT_^1^	*N*_PT_^2^	*N*_Cal_^3^	Defect	Comments
**Increasing descriptors**									
1	HALO00000000	6.1031	6.6098	8.8583	144	146	123	0	Absence of halogen
2		8.633	7.0133	3.7267	163	152	102	0	
3		9.6479	3.4235	2.7648	151	143	119	0	
1	PT3-H…2…	0.73	0.708	0.4981	128	142	115	0.0004	The presence of the path of length 3 equal to 2 for hydrogen atom
2		0.1459	0.6061	1.1117	153	138	97	0.0002	
3		0.4238	0.3956	1.0186	137	139	112	0.0003	
1	[*xyzyx*0]…	2.0073	1.413	2.1025	134	140	110	0.0003	The absence of symmetry *xyzyx*
2		1.1165	2.7083	1.7976	146	146	95	0.0003	
3		2.2966	2.5602	2.774	137	137	110	0.0003	
1	EC1-H…3…	1.4729	0.8766	1.6115	87	103	71	0.001	Morgan extended connectivity of first-order for hydrogen atom as 3
2		1.7619	1.0208	1.8646	106	101	65	0.0002	
3		1.2874	1.159	1.0864	89	101	70	0.0009	
1	PT2-H…2…	0.9081	1.1622	1.1356	87	103	71	0.001	The presence of the path of length 2 equal to 2 for hydrogen atom
2		1.3099	2.7157	0.7392	106	101	65	0.0002	
3		1.4278	1.6415	1.3682	89	101	70	0.0009	

**Decreasing descriptors**									
1	(………	−0.0011	−0.5156	−0.1639	138	142	122	0.0002	Presence of branching
2		−0.2948	−0.0691	−0.1499	158	147	98	0	
3		−0.1117	−0.4944	−0.4026	146	138	118	0.0001	
1	EC0-H…1…	−0.2507	−0.2526	−0.0614	145	153	115	0.0002	Morgan extended connectivity of zero-order for hydrogen atom as 1
2		−0.1227	−0.3508	−0.8636	163	144	104	0.0001	
3		−0.3708	−0.3673	−0.1312	165	163	86	0.0002	
1	EC1-H…2.	−0.405	−0.4271	−0.3171	128	139	120	0.0004	Morgan extended connectivity of first-order for hydrogen atom as 2
2		−0.5502	−0.7819	−0.0901	153	144	92	0.0002	
3		−0.4393	−0.4116	−0.1462	136	135	116	0.0004	
1	EC0-O…2…	−0.9507	−0.2171	−0.2003	133	139	119	0.0002	Morgan extended connectivity of zero-order for oxygen atom as 2
2		−0.3374	−0.3291	−0.2217	155	143	94	0.0001	
3		−0.8798	−0.5985	−0.0551	3288	3288	2640	0.0002	
1	PT2-O…2…	−0.2695	−0.3376	−0.1982	2472	1704	0.024	0.0003	The presence of the path of length 2 equal to 2 for oxygen atom
2		−0.7329	−0.4182	−0.494	151	139	94	0.0001	
3		−0.1138	−0.4556	−0.5146	137	136	115	0.0003	
1	NNC-O…202	−0.3491	−0.9969	−0.5012	123	138	115	0.0005	The Nearest Neighbor Codes for Oxygen (NNC-O) equal to 202
2		−0.6164	−0.7005	−0.4575	148	136	93	0.0001	
3		−0.5194	−0.488	−0.7235	131	136	112	0.0004	
1	VS2-H…3…	−0.3884	−0.2012	−0.2146	117	134	112	0.0006	Valence shell of second order equal to 3 for hydrogen atoms
2		−0.3008	−0.7597	−0.3096	142	133	90	0.0001	
3		−0.1958	−0.4599	−0.2253	126	131	109	0.0004	
1	[N+]……	−0.52	−0.6575	−0.1623	118	130	116	0.0007	Positively charged nitrogen-containing structural feature
2		−0.1057	−0.2152	−0.0743	144	137	86	0.0003	
3		−0.5138	−0.3283	−0.0508	126	126	112	0.0006	

According to the presented data, the structural attributes associated with increased endpoint include: absence of halogen (HALO00000000), the presence of the path of length 3 equal to 2 for hydrogen atom (PT3-H…2…) the absence of symmetry *xyzyx* ([*xyzyx*0]…), Morgan extended connectivity of first-order for hydrogen atom as 3 (EC1-H…3…), and the presence of the path of length 2 equal to 2 for hydrogen atom (PT2-H…2…), while promoters associated with log *E*_50_ decrease were: presence of branching ((………), Morgan extended connectivity of zero-order for hydrogen atom as 1 (EC0-H…1…), Morgan extended connectivity of zero-order for oxygen atom as 2 (EC0-O…2…), the presence of the path of length 2 equal to 2 for oxygen atom (PT2-O…2…), the Nearest Neighbor Codes for Oxygen (NNC-O) equal to 202 (NNC-O…202), valence shell of second order equal to 3 for hydrogen atoms (VS2-H…3…), and positively charged nitrogen-containing structural feature ([N+]……).

### 3.5. Comparison of the results with published models

Researchers have established numerous predictive parameters based on the molecular structures and properties by correlating impact sensitivities such as oxygen balance, molecular electronegativity, partial atomic charge, electrostatic potentials, bond dissociation energy and vibrational modes.^13,15,19,51–55^ In addition, many QSPR models and machine learning (ML) methods have been developed for this property.^2–4,7–9,12,14,56^ However, some focused only on specific types of energetic compounds, and most used small datasets, with the exception of the model reported by Marrs *et al.*^28^

A meaningful comparison requires consideration of dataset characteristics, descriptor representations, and validation procedures, in addition to reported statistical parameters. Prior studies have typically focused on particular compound classes-nitroaliphatic, nitroaromatic, or nitramine-whereas the present dataset comprises 515 energetic compounds covering a broader chemical space. Such differences in composition and structural diversity may influence reported performance and preclude direct comparison of numerical statistical parameters across studies.

Earlier QSPR studies employed descriptors based on physicochemical properties, semi-empirical quantum-chemical calculations, molecular topology, or three-dimensional representations, while others adopted ML algorithms such as artificial neural networks and related nonlinear techniques. Although these approaches offer flexible representations of structure–property relationships, their performance depends on descriptor quality, model architecture, training procedure, and validation design. In contrast, the present study uses hybrid descriptors derived from SMILES and hydrogen filled graph (HFG), with correlation weights optimized *via* the Monte Carlo procedure, thereby minimizing the need for external descriptor calculations and enabling investigation of individual structural attributes.

A further distinction concerns interpretability. Models based on quantum-chemical descriptors or flexible ML architectures may yield useful predictions but often require additional analysis to elucidate the structural origin of the endpoint. Here, SMILES–HFG descriptors and analysis of structural attributes associated with increases or decreases in log *E*_50_ provide a basis for qualitative mechanistic interpretation; however, these contributions should be considered model-dependent associations rather than independent proof of the underlying physical mechanism of impact sensitivity.

For reference, Marrs *et al.* analyzed a data set of 1533 observations of 515 energetic molecules using 156 descriptors. The data included 34 molecules with 1052 repeat observations and 481 molecules with a single observation. They applied random forest (RF) method to model log *E*_50_ as linear and nonlinear functions of the molecular descriptors. They found that the model using variable selection with random forests (VSURF; 19 descriptors) outperformed least absolute shrinkage and selection operator (LASSO) and RF methods, achieving the value of 
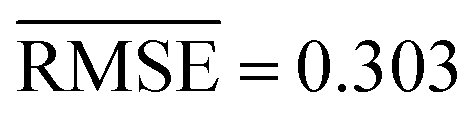
 for the selected method was corresponded to an approximate coefficient of determination *R*^2^ ≈ 0.64.

In our earlier report,^57^ we developed 12 QSPR models to predict the impact sensitivity of 404 nitroenergetic compounds with the same software (CORAL) using four target functions *i.e.* TF0 without IIC or CII, TF1 without IIC, TF2 without CII and TF3 with IIC and CII.·In that study, we compared several QSAR and QSPR models for predicting the impact sensitivity of energetic materials with the proposed model. In contrast, the present study employs an expanded dataset comprising 515 compounds. However, a rigorous comparison of the statistical performance of these models remains challenging because of differences in dataset composition, molecular coverage, and the allocation of compounds between the training and test sets. Despite this limitation, the proposed model offers several notable advantages.

The previously reported models were developed using relatively smaller datasets (*n* < 404), whereas the present study utilizes the large dataset, comprising 515 energetic compounds. The use of a larger and more diverse dataset provides broader molecular coverage and can help reduce the risk of obtaining inflated *R*^2^ values, thereby highlighting the limitations of relying on *R*^2^ alone as a basis for model comparison. In most previous studies, QSPR models were developed using two data subsets, namely training and test sets, with the notable exceptions of the models reported by Wang *et al.*^2,13^ and in our previous study.^57^ In contrast, the QSPR models developed in the present work were constructed using four distinct subsets: active training, passive training, calibration, and validation sets, allowing their robustness and predictive performance to be evaluated from multiple perspectives. Moreover, unlike previous approaches that generally relied on multiple molecular descriptors, the present models employ a single hybrid descriptor, DCW, providing a more concise representation of the molecular structure–property relationship. Furthermore, while the previously reported models were evaluated primarily using *R*^2^ and *R*_test_^2^, the present study adopts a more comprehensive set of statistical criteria, including *R*^2^, IIC, CII, CCC, *Q*^2^, *Q*_F1_^2^, *Q*_F2_^2^, *Q*_F3_^2^, MAE, *r*_m_^2^, *r*_m_^2^_,_
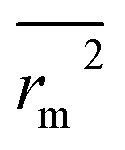
 , RMSE, CR^2^p, F, and Y test. This comprehensive evaluation framework enables a more rigorous assessment of the robustness, reliability, and predictive performance of the proposed models.

## 4. Conclusions

In the present study, three QSPR models were developed to estimate the log *E*_50_ of 515 energetic compounds using hybrid DCW descriptor computed by CORAL software. These models were constructed *via* the balance of correlation algorithm with IIC and CII. The most reliable improvement in predictive potential was achieved through the combined use of IIC and CII. All developed models demonstrated satisfactory statistical robustness and predictive reliability, confirming their applicability to log *E*_50_ prediction. Notably, the model generated using split 1 provided the best validation performance among the developed models, yielding the highest correlation coefficient for the validation set (*R*_VAL_^2^ = 0.7106). The mechanistic interpretation of the models was performed through examination of the correlation weights of different molecular features obtained from several Monte Carlo optimization runs.

## Conflicts of interest

The authors declare that they have no known competing financial interests or personal relationships that could have appeared to influence the work reported in this paper.

## Supplementary Material

RA-016-D6RA07617C-s001

## Data Availability

The authors declare that the data supporting the findings of this study are available within the paper and its supplementary information (SI) files. Should any raw data files be needed in another format they are available from the corresponding author upon reasonable request. Supplementary information: the dataset of energetic compounds, their SMILES representations, details of the dataset partitioning, and additional results of the developed QSPR models. See DOI: https://doi.org/10.1039/d6ra07617c.
